# Improving awareness of kidney function through electronic urine output monitoring: a comparative study

**DOI:** 10.1186/s12882-022-03046-5

**Published:** 2022-12-27

**Authors:** Omar Murad, Daniel F Orjuela Cruz, Aliza Goldman, Tal Stern, Peter Vernon van Heerden

**Affiliations:** 1 The Hadassah Medical Center, Jerusalem, Israel; 2 Clinical Research Department, RenalSense Ltd, 3 Hamarpe St, Har Hotzvim, Jerusalem, Israel

**Keywords:** Electronic urine output monitoring, Oliguria, Acute kidney injury, Length of stay, Serum creatinine

## Abstract

**Background:**

The current classification for acute kidney injury (AKI) according to the Kidney Disease: Improving Global Outcomes (KDIGO) criteria integrates both serum creatinine (SCr) and urine output (UO). Most reports on AKI claim to use KDIGO guidelines but fail to include the UO criterion. It has been shown that patients who had intensive UO monitoring, with or without AKI, had significantly less cumulative fluid volume and fluid overload, reduced vasopressor use, and improved 30-day mortality. We examined whether real-time monitoring of this simple, sensitive, and easy-to-use biomarker in the ICU led to more appropriate intervention by healthcare providers and better outcomes.

**Methods:**

RenalSense Clarity RMS Consoles were installed in the General ICU at the Hadassah Medical Center, Israel, from December 2019 to November 2020. The Clarity RMS system continuously and electronically monitors UO in real-time. 100 patients were randomly selected from this period as the study group (UO_elec_) and compared to a matched control group (UO_manual_) from the same period two years earlier. To test whether there was an association between oliguric hours and fluid treatment in each group, the correlation was calculated and analyzed for each of the different UO monitoring methods.

**Results:**

Therapeutic intervention: The correlation of the sum of all oliguric hours on Day 1 and 2 with the sum of any therapeutic intervention (fluid bolus or furosemide) showed a significant correlation for the study group UO_elec_ (*P* = 0.017). The matched control group UO_manual_ showed no such correlation (*P* = 0.932). Length of Stay (LOS): Median LOS [IQR] in the ICU of UO_elec_ versus UO_manual_ was 69.46 [44.7, 125.9] hours and 116.5 [62.46, 281.3] hours, respectively (*P* = 0.0002).

**Conclusions:**

The results of our study strongly suggest that ICU patients had more meaningful and better medical intervention, and improved outcomes, with electronic UO monitoring than with manual monitoring.

**Supplementary Information:**

The online version contains supplementary material available at 10.1186/s12882-022-03046-5.

## Introduction

The goal of intensive and automated monitoring of vital signs and physiological parameters in the intensive care unit (ICU) is to provide timely information and support appropriate intervention by healthcare providers [1]. Additionally, databases of these vital signs are crucial for retrospective studies of illness progression and to bring about protocol changes for better patient-directed care [2]. Unfortunately, urine output (UO) remains one of the few parameters monitored manually.

Over the past two decades, UO has been validated as a vital biomarker for acute kidney injury (AKI) [3–5]. The current classification for AKI recommended by the Kidney Disease: Improving Global Outcomes (KDIGO) Clinical Practice Guideline for AKI, integrates both serum creatinine (SCr) and UO in their severity score for AKI [6]. The KDIGO guidelines suggest use of a “bundle” of supportive measures in patients at high risk for AKI. This bundle includes, *inter alia*, maintenance of volume status and monitoring of SCr and UO [6]. UO monitoring is necessary to detect decreases in renal blood flow and/or a decline in renal perfusion pressure [7].

In a study examining UO monitoring, with or without AKI, patients who had intensive UO monitoring (defined as a manual measurement at least every 3 h), had significantly less cumulative fluid volume and fluid overload. They were also significantly less likely to receive vasopressors over the first 72 h of their ICU stay. Intensive UO monitoring was shown to be independently associated with improved 30-day survival among patients developing AKI [8].

A recent study reviewed surgical ICU patient UO monitoring protocols. Only 66% showed any UO monitoring as part of their routine care [7]. Most reports on AKI claim to use KDIGO guidelines but use only the SCr criterion in their definition of AKI, i.e., they fail to include the UO criterion [9]. UO monitoring is a simple, sensitive, and easily available biomarker. Emphasizing its strict use could allow for earlier detection of AKI [10]. Inconsistent UO measurements and the nature of manual monitoring alters the reported incidence and may delay the diagnosis of AKI [9, 11–13]. This can lead to underestimation of the association between AKI and ICU mortality [14]. In a recent review of the impact of integrating biomarkers for patient care for AKI, the authors presented evidence of improvement of patient outcomes when close monitoring of functional biomarkers for AKI (such as SCr and UO) was performed. Specifically, when using electronic health monitoring and real-time data, these biomarkers, used alongside patient risk factors and renal reserve tests, help target care bundles to optimize patient care [15].

We set out to assess the effects of a change in the routine care of ICU patient UO monitoring. We designed a comparison study to evaluate the consequent change in the awareness of renal function by the medical staff in a general ICU following installation of an electronic monitoring system on every ICU bed. “Awareness” was evaluated by examining outcomes such as timely UO reporting, response to treatment for oliguria, physician daily reports, and length of stay (LOS). These objective measures were compared to a matched control group two years prior to the installation of the electronic UO monitoring system.

## Methods

### Study design

#### The RenalSense Clarity RMS device

**Fig. 1:**
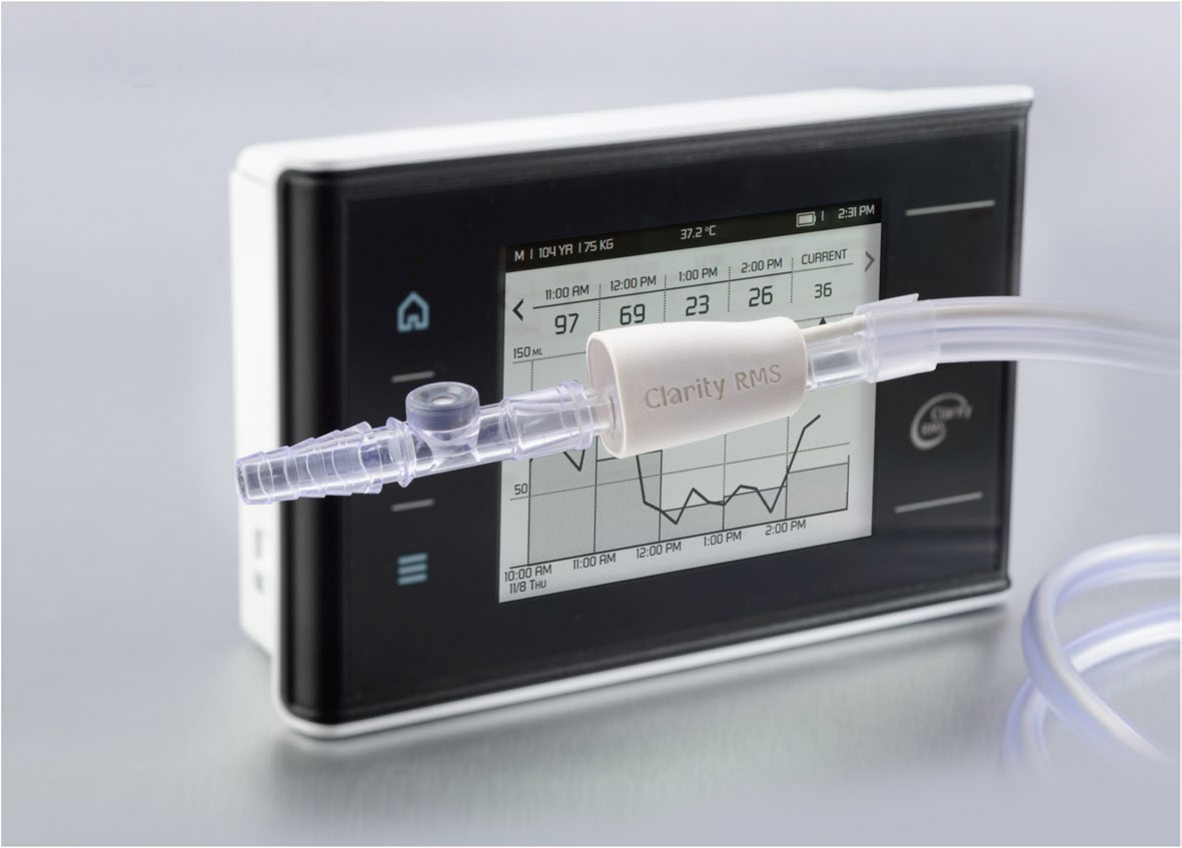
RenalSense Clarity RMS™ Console and Sensor Kit monitors urine output in real-time

For this study, the RenalSense Clarity RMS Console (Fig. 1) for electronic monitoring of UO was installed on every bed in the general ICU at Hadassah Medical Center in Israel from November 2019 to November 2020. Local IRB approval was obtained from the Helsinki Ethics Committee at the Hadassah Medical Center. All methods were carried out in accordance with relevant guidelines and regulations. Because this trial analyzed department-wide use of a non-invasive device, and compared the data to retrospective electronic records, informed consent was waived by the Hadassah Medical Center Helsinki Ethics Committee. Using the RenalSense Clarity RMS Sensor Kit that connects to a standard in-dwelling Foley urinary catheter, the system continuously monitors UO in real-time. Its technology is described elsewhere [16].

Nurses were trained for four weeks on use of the device before the study began. A daily average of 70% of catheterized ICU patients were connected to the system. The display on the Console shows hourly UO measurements, updated every 15 min. Below the table of numerical output, a graph displays 15-min UO. Staff can pan back to review the patients’ UO history (Fig. 1).

#### Study group

One hundred twelve catheterized patients connected to the electronic UO monitoring system were randomly chosen between December 23^rd^, 2019, to November 1^st^, 2020. Of those, 100 patients that could be paired with a matched control group were included in the study group (**UO**_**elec**_**)**. *Inclusion criteria:* Patients ≥ 18 years of age. *Exclusion criteria:* patients who were discharged or died within 24 h in the ICU; patients on dialysis; and pregnant women.

#### Matched control group

Installation of a new electronic UO monitoring device in the department would affect a concurrent control group as well as the study group. Therefore, the study was divided into two stages: (1) prior to installation and (2) after installation. A retrospective matched control group of 100 catheterized patients (**UO**_**manual**_) was selected in a parallel time period two years prior to the installation (December 23, 2017, to November 1, 2018), using the same inclusion and exclusion criteria as the study group.

Patient recruitment was done by a neutral party of two medical residents, not involved in treating the ICU patients enrolled in either group. Patient recruitment for the control group prioritized the matching in a top-down manner in the following order:

1. Matched admission cause.
2. Matched pairs at admission were categorized as observational/interventional treatment of said cause of admission in the ICU.
3. Patient comorbidities were matched after steps 1–2 based on < 2 or ≥ 2 comorbidities for acute kidney injury.
4. Patient’s first 24 h in the ICU were matched for progression of illness as stable/unstable and mechanical ventilation Y/N.
5. After steps 1–4, matching for age was initially set for ≤  ± 10 years difference between matched pairs. However, due to the limited patient pool, this was increased to ≤  ± 12 years.
6. Matching according to sex after steps 1–5 was placed as the lowest priority due to the limited patient pool. This matching was achieved in 71% of patients.

Patients were de-identified and all data was analyzed and compared between the two groups by unbiased, outsourced biostatisticians.

#### Patient demographics

Patient information recorded included: age, weight, sex, baseline SCr, primary diagnosis, comorbidities, need for mechanical ventilation, use of vasoactive drugs and APACHE II scores in the first 24 h in the ICU.

#### Nursing-related renal assessment

*UO measurements:* Hourly UO measurements were retrieved from nursing records of patient files and compared between **UO**_**elec**_, and **UO**_**manual**_. Oliguric hours were defined as UO < 0.5 ml/kg/hr. *Patient UO monitoring:* The percent of time without hourly UO records was analyzed from admission and up to the first seven days of ICU hospitalization. ‘Missing hours’ refers to the hours a patient was catheterized, in the department, and UO was not charted in their file. Hours that patients were transferred for a test such as an MRI or CT, or other clinically valid reasons outside the department, were not included in the analysis of missing hours. *Manual hourly output reporting time:* Due to the large number of hours required to analyze hourly UO reporting, 25 of the 100 patients were randomly selected from the **UO**_**manual**_ group. The *time to nurses reporting* for manual measurements was based on the time stamp recorded in the computer when the UO was charted, versus the timeslot in which the observed UO was inserted. This analysis was not relevant for the study group, UO_**elec**_, since measurements are automatically recorded on the hour.

#### Physician-related renal assessment

*Physician daily reports:* Assessment of physician daily reporting included their mention of renal parameters, related treatment, and patient fluid status. The renal parameters included: SCr, UO, and fluid balance*-* either as the actual measurement (e.g., ‘UO 200 ml in the past 24 h’), or as a descriptive measurement (e.g., ‘increasing SCr’, or ‘positive fluid balance’, etc.); renal function described as: ‘normal’, ‘injury’, ‘failure’, ‘stable’ or ‘other’. Diuretic and fluid bolus and patient fluid status were noted as part of their treatment and follow-up in the report. The analysis compared the frequency of reporting these parameters in the **UO**_**elec**_ versus the **UO**_**manual**_ groups for up to the first seven days of their ICU stay. The comparison of each parameter was included individually as well as a summary comparison of any renal parameter reported. SCr was analyzed for 7 days as per the KDIGO guidelines for identifying AKI with this criterion [6].

#### Outcomes

*Therapeutic intervention:* The amounts of fluid bolus and diuretic treatment administered during the first 48 h in the ICU were analyzed. To test whether there was an association between oliguric hours and fluid treatment in each group, the correlation was calculated and analyzed for each of the different UO monitoring methods. Diuretic and bolus administration treatments were correlated to patient oliguric hours monitored within the first 48 h after admission using the KDIGO guidelines for defining AKI according to UO [6].

*Length of stay* in the ICU was compared between the **UO**_**elec**_ and **UO**_**manual**_ groups.

#### Statistical analysis

Statistical analysis was performed using R 3.5.0. Continuous variables were summarized by a mean, standard deviation, median, IQR (intra-quartile range), minimum, and maximum, and categorical variables by a count and percentage. 95% Confidence intervals were provided where relevant. For comparison of continuous variables, the two-sample t-test or the Wilcoxon rank-sum test was used, as appropriate. For comparison of proportions (categorical variables), the Wald test was used. For correlation evaluation test, Pearson correlation coefficient or Spearman's rank correlation coefficient was used. Pearson's correlation between oliguric hours and treatment on each of the first two days in the ICU was tested for the **UO**_**elec**_ and **UO**_**manual**_ data separately. This was repeated for each day and treatment separately, as well as a combination of any treatment versus any oliguria. Lengths of stay in the ICU are presented using mean (SD) and median (IQR). Comparison between the control and the study groups was done with Wilcoxon rank-sum test. No corrections for multiple comparisons were performed. A time-to-discharge analysis was performed, using Kaplan–Meier curves and log rank test. All statistical tests were two-sided. The required significance level of findings was equal to or lower than 5%. Nominal P-values are presented. As a sensitivity analysis, all analyses were repeated for the patients enrolled in the study before and after the COVID-19 pandemic began, and their matched subjects from parallel time frames in 2018, to assure similar trends, regardless of pandemic-related effects.

## Results

### Patient demographics

The study group, **UO**_**elec**_ and the matched control group, **UO**_**manual**_, both comprised 100 patients. The majority admission cause comprised 48 surgical patients in each group. Other causes of admission included neurological/neurosurgical, trauma, burns, and other causes. There were 38 versus 29 females respectively, in **UO**_**elec**_ and **UO**_**manual**_ groups. Median [IQR] ages were similar 65.6 [45.6, 75.2] and 67.0 [47.8, 74.9] years, for **UO**_**elec**_ and **UO**_**manual**_ groups, respectively. Median [IQR] APACHE scores were 20 [16, 25] and 21 [14, 28] for the **UO**_**elec**_ and **UO**_**manual**_ groups, respectively (Table 1).

**Table 1:** patient information

	**UO**_**manual**_		**UO**_**elec**_		***P*-value**
Cause of admission					
Surgical	48	48%	48	48%	
Neurological/surgical	13	13%	13	13%	
Sepsis/septic shock	11	11%	11	11%	
Trauma	14	14%	14	14%	
Burn trauma	4	4%	4	4%	
Other	10	10%	10	10%	
Sex- n (%)					
F	29	29.0%	38	38.0%	0.1771
M	71	71.0%	62	62.0%	
Age- Mean (SD)	60.9 (20.0)		61.0 (20.6)		0.9708
Median (IQR)	67.0 (47.8, 74.9)		65.6 (45.6, 75.2)		
Range (Min, Max)	(18.9, 93.9)		(18.5, 99.7)		
Weight- Mean (SD)	77.4 (14.7)		76.87 (15.0)		0.7624
Median (IQR)	75.0 (70.0, 85.0)		77.5 (67.5, 85.5)		
Range	(48.0, 120.0)		(50.0, 110.0)		
APACHE- Mean (SD)	21.4 (9.1)		20.4 (7.5)		0.4021
Median (IQR)	21 (14, 28)		20 (16,25)		
Range	(2, 42)		(0, 43)		
APACHE ≥ 25	35	35%	30	30%	0.4506
Receiving vasopressors Y/N- n (%)					
N	42	42.0%	48	48.0%	0.3940
Y	58	58.0%	52	52.0%	

### Patient UO monitoring

During the first four ICU days, an average of 5.92% (± 10.8%) of hourly UO reports were missing in the **UO**_**manual**_ group versus 0.69% (± 1.6%) in the **UO**_**elec**_ group, (*P* < 0.0001) (Table 2).

**Table 2:** Percent missing of hourly UO reporting

		**UO**_**manual**_			**UO**_**elec**_		
Relevant monitoring days	N	Mean (SD) (%)	Median (IQR) (%)	N	Mean (SD) (%)	Median (SD) (%)	*P*-value
Monitored for < 4 days	50	5.92 (10.8)	3.33 (1.33- 6)	61	0.69 (1.6)	0 (0- 0)	< .0001
Monitored for ≥ 4 days	50	3.17 (2.25)	2.9 (1.84- 4.43)	39	0.47 (0.86)	0 (0- 0.69)	< .0001

### Manual hourly output reporting time

Twenty five patients were selected with an aggregate total of 1214 hourly **UO**_**manual**_ recorded measurements. The average delay time to reporting was 39.54 min, with a 95% CI of (37.02- 42.05). A delay of an hour or more were reported in 24.2% of these measurements. There was no delay in **UO**_**elec**_ measurements.

### Daily physician reporting

**Fig. 2:**
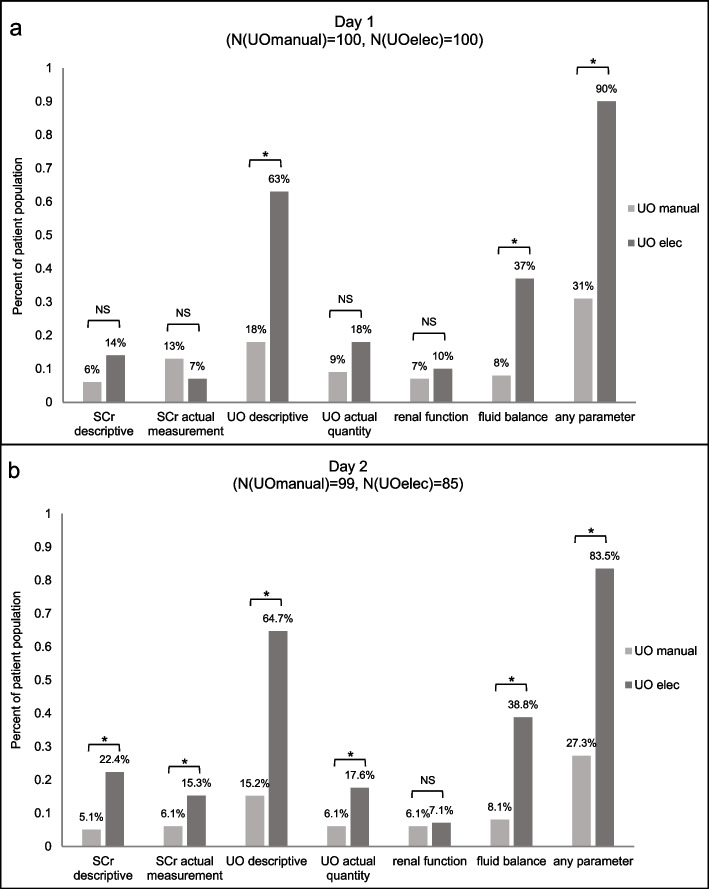
Comparison between the study group and the matched control of renal parameters recorded in physician daily reports. **a** Day 1 of ICU admission, **b** Day 2 of ICU admission

**Fig. 3:**
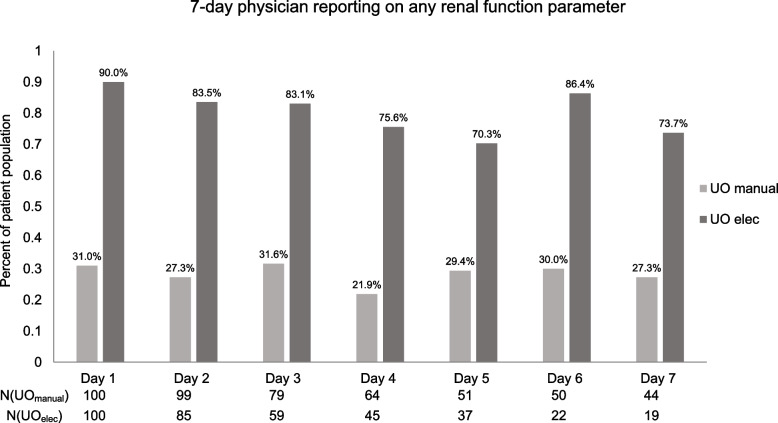
Comparison of the physician daily reports over 7 days for any of the listed parameters of renal function

There was a statistically significant greater daily reporting of any renal function parameter in the **UO**_**elec**_ group as compared to the **UO**_**manual**_ for all ICU hospitalization days (*P* < 0.0001) (Table 3, Fig. 2a and b, supplementary Fig. 1a-e). There was also a statistically significant greater reporting for *UO descriptive* and *fluid balance* across almost all days (Table 3) (Figs. 2a,b and 3 and supplementary Fig. 1a-e). Between day 2 to day 3 there was a statistically significant improvement in the **UO**_**elec**_ group reporting any renal parameter mentioned except for *renal function* in which there was no change. (Table 3). (Figs. 2a,b and 3, and supplementary Fig. 1a-e).

**Table 3:** Physician reporting on renal parameters

	**Day 1**			**Day 2**			**Day 3**			**Day 5**			**Day 6**			**Day 7**		
	**(N( UO**_**manual**_**) = 100,**			**(N( UO**_**manual**_**) = 99,**			**(N( UO**_**manual**_**) = 79,**			**(N( UO**_**manual**_**) = 51,**			**(N( UO**_**manual**_**) = 50,**			**(N( UO**_**manual**_**) = 44,**		
	**N( UO**_**elec**_**) = 100)**			**N( UO**_**elec**_**) = 85)**			**N( UO**_**elec**_**) = 59)**			**N( UO**_**elec**_**) = 37)**			**N( UO**_**elec**_**) = 22)**			**N( UO**_**elec**_**) = 19)**		
	**UO**_**manual**_	**UO**_**elec**_	*P*-value	**UO**_**manual**_	**UO**_**elec**_	*P*-value	**UO**_**manual**_	**UO**_**elec**_	*P*-value	**UO**_**manual**_	**UO**_**elec**_	*P*-value	**UO**_**manual**_	**UO**_**elec**_	*P*-value	**UO**_**manual**_	**UO**_**elec**_	*P*-value
**SCr descriptive**	6.0%	14.0%	0.0585	5.1%	22.4%	0.0007	8.9%	18.6%	0.1050	7.8%	18.9%	0.1412	14.0%	22.7%	0.3948	9.1%	5.3%	0.5705
**SCr actual measurement**	13.0%	7.0%	0.1568	6.1%	15.3%	0.0453	5.1%	15.3%	0.0562	7.8%	5.4%	0.6461	6.0%	9.1%	0.6597	9.1%	21.1%	0.2504
**UO descriptive**	18.0%	63.0%	< 0.0001	15.2%	64.7%	< 0.0001	20.3%	54.2%	< 0.0001	11.8%	59.5%	< 0.0001	18.0%	50.0%	0.0093	15.9%	47.4%	0.0161
**UO actual quantity**	9.0%	18.0%	0.0618	6.1%	17.6%	0.0163	2.5%	23.7%	0.0004	7.8%	5.4%	0.6461	8.0%	18.2%	0.2657	11.4%	15.8%	0.6477
**Renal function**	7.0%	10.0%	0.4471	6.1%	7.1%	0.7859	7.6%	5.1%	0.5444	7.8%	0.0%	0.0402	4.0%	4.5%	0.9173	6.8%	0.0%	0.0777
**Fluid balance**	8.0%	37.0%	< 0.0001	8.1%	38.8%	< 0.0001	11.4%	35.6%	0.0010	11.8%	32.4%	0.0229	10.0%	22.7%	0.2024	11.4%	36.8%	0.0387
**Any renal parameter mentioned**	31.0%	90.0%	< 0.0001	27.3%	83.5%	< 0.0001	31.6%	83.1%	< 0.0001	29.4%	70.3%	< 0.0001	30.0%	86.4%	< 0.0001	27.3%	73.7%	0.0003

### Fluid bolus and diuretic administration

A negative correlation was found between furosemide treatment and fluid bolus (i.e., if the patient received one of the treatments, they are less likely to receive the other). Oliguria on Day 1 was strongly correlated with oliguria on Day 2 in both the UO_elec_ and UO_manual_ groups (*r* = 0.548 and 0.549, respectively). The correlation between all oliguric hours on Day 1 and 2 with any treatment (bolus or furosemide) for the UO_elec_ group showed a significant correlation, while the UO_manual_ group showed no such correlation (*r* = 0.246 and *P* = 0.017, and *r* = 0.009 and *P* = 0.932, respectively) (Table 4).

**Table 4:** Correlation between oliguric hours and related treatment (*indicates significance)

	**UO**_**manual**_	**UO**_**elec**_
Correlation of Oliguria Day 1, Treatment Day 1	0.081	0.123
Correlation of Oliguria Day 2, Treatment Day 2	-0.032	0.281*
Summary correlation of Day 1 and Day 2		
Pearson’s Correlation	0.009	0.246*
*P*-value	0.932	0.017*

### Length of stay

**Fig. 4:**
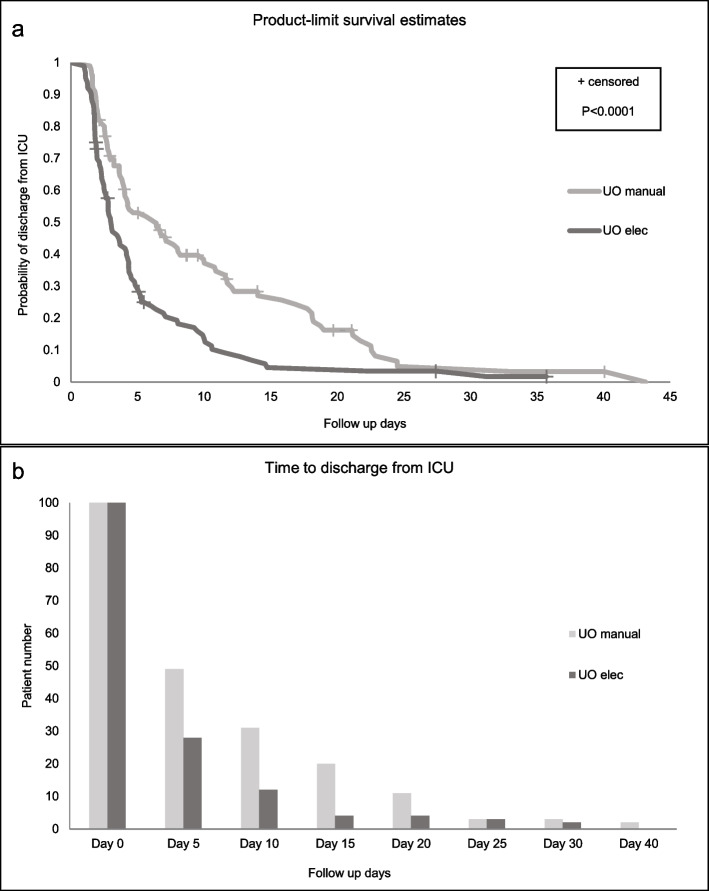
Discharge from the ICU. **a** Kaplan–Meier curve comparing probability of discharge between the study group and the matched control. **b** Comparison of time to discharge between the study group and the matched control

Median [IQR] LOS in the ICU of **UO**_**elec**_ group versus **UO**_**manual**_ group was 69.46 [44.7, 125.9] hours and 116.5 [62.46, 281.3] hours, respectively (*P* = 0.0002). By Day 5, 69 out of the 100 patients in the **UO**_**elec**_ were released from the ICU and 3 died, as compared to 47 released out of 100 patients in the **UO**_**manual**_ group and 5 deaths. The length of stay was significantly shorter in the **UO**_**elec**_ group versus the **UO**_**manual**_ group (*P* < 0.0001) ( Fig. 4a and b).

## Discussion

Acute kidney injury has been reported in up to 24% of trauma patients, 30% of patients after cardiac surgeries, up to 42% of patients hospitalized with severe acute respiratory syndrome coronavirus-2 (SARSCoV-2) and over 50–75% of all ICU patients [17–21]. UO has been shown to be an early indicator of AKI and management of AKI progression can be implemented using intensive UO monitoring [7, 9, 12, 15]. Our study comparison highlighted the difficulty in obtaining consistent and reliable hourly measurements of UO when patients are monitored manually. Ultimately, this erratic UO monitoring may interfere with timely identification of oliguria and patients at risk for AKI [22].

Our study showed that ICU patients electronically monitored for UO received treatment with furosemide and fluid bolus that was significantly better correlated to patient urine output than the manually monitored patient group. This, together with the significant increase in physician daily reporting of renal parameters in our study group as compared to the matched control, has shown an important advantage to electronic real-time UO monitoring as the standard of care.

Predicting those at risk for AKI is a prominent goal to improve patient care and lower healthcare costs. Some studies point to the physiological causes of oliguria as an indication that UO is too sensitive a biomarker for AKI [23]. However, many other studies have shown that oliguria alone, even in the absence of a rise in SCr, identifies patients who have worse outcomes such as increased LOS, AKI severity, dialysis, and hospital costs [3, 5, 24–26]. Our study has shown that the study group had a significantly shorter LOS as compared to the matched control. This significant difference indicates the impact that intensive real-time UO monitoring, and consequent intensive monitoring of all renal parameters, has on reduction of ICU LOS.

Machine learning (ML) using prediction models has become more popular in healthcare environments. These have the goal of identifying digital patterns to improve accuracy in clinical diagnosis and, ultimately, to improve patient care. We are in an era of digitized monitoring of all physiological parameters and this aids development of algorithms for prediction models. For example, recent research has demonstrated that machine learning can identify septic patients at risk of developing oliguria after fluid administration [27]. Prediction models such as these may provide clinicians with a tool to identify fluid non-responders, minimize damage and implement more effective fluid resuscitation protocols [27]. Missing and inaccurate data, such as we have shown occurs with manual UO measurements, interferes with reliable reporting in patient electronic health records and the accuracy of models based on them [22, 28]. The transition of urine measurement from the manual era to the automated digital one will promote implementation of better ML models.

Fluid overload has been shown to be an independent risk factor for AKI in ICU patients, in patients with sepsis, and patients after cardiovascular surgery. We have shown the improvement of awareness to patients’ renal status when there is reliable monitoring of UO displayed as a continuous trend for use in a general ICU. Intensive monitoring of UO can guide fluid resuscitation treatment and help physicians intervene early and prevent unnecessary fluid overload [7, 9, 13, 15].

### Study limitations

The temporal gap between the study cohort and the comparison group may be viewed as a study limitation, however, we can confirm that there were no changes in clinical practice and protocols, nor in the approach to fluid management during these two periods. We also acknowledge that the interventions chosen and recorded in the study (fluid administration and/or diuretics) in response to observed oliguria may not have been either “positive”, or “negative” in each individual case. The focus of this study, though, was to assess the extent to which awareness of kidney function, and treatment in response to such awareness, was affected. Since treatment was chosen by qualified medical experts according to hospital protocols, assessing their protocols was beyond the scope of this study.

Additionally, our study was a single center trial. Further studies using electronic UO monitoring should be developed for larger, multi-centered trials to analyze the impact of such monitoring on these and other outcomes such as ICU mortality and one-year-mortality rates. The impact of the use of the monitoring system was analyzed retrospectively in our study. The improvement of volume status, daily fluid balance trends, and fluid overload, blood pressure and use of vasopressors, should be further explored in prospective fluid resuscitation trials using real-time UO monitoring. Further studies with this monitoring should also include rates of incidence, severity, and resolution of acute kidney injury.

## Conclusion

The plethora of data from electronic health records (EHR) and the computing power of modern-day systems provide enormous advantage to the machine learning models for predicting disease in real-time [29]. The current clinical standard of manual UO monitoring is a continuing limitation for implementation of these ML models. Furthermore, our findings show the importance of continuous real-time UO monitoring including its contribution to better awareness of patient renal parameters. Better awareness supports the implementation of goal-directed treatment that ultimately leads to better ICU outcomes.

## Supplementary Information

**Additional file 1:Supplementary Fig 1.** Comparison between the study group and the matched control of renal parameters recorded in physician daily reports a. Day 3 in ICU b. Day 4 in ICU c. Day 5 in ICU d. Day 6 in ICU e. Day 7 in ICU.

## Data Availability

The datasets used and/or analysed during the current study are available from the corresponding author on reasonable request.
