# Supplementary material for: Improving awareness of kidney function through electronic urine output monitoring: a comparative study
Source: BMC Nephrol. 2022 Dec 27;23:412. doi: 10.1186/s12882-022-03046-5 (PMC9792308; doi:10.1186/s12882-022-03046-5)
Supplement: Supplementary file 1 — Additional file 1:Supplementary Fig 1. Comparison between the study group and the matched control of renal parameters recorded in physician daily reports a. Day 3 in ICU b. Day 4 in ICU c. Day 5 in ICU d. Day 6 in ICU e. Day 7 in ICU. [file 12882_2022_3046_MOESM1_ESM.docx]

Table of Contents

**Supplementary Fig 1a and 1b**2

**Supplementary Fig 1c and 1d**3

**Supplementary Fig** **1e**4

Supplementary figures legend

Supplementary Fig 1: Comparison between the study group and the matched control of renal parameters recorded in physician daily reports a. Day 3 in ICU b. Day 4 in ICU c. Day 5 in ICU d. Day 6 in ICU e. Day 7 in ICU

Supplementary Fig 1a.


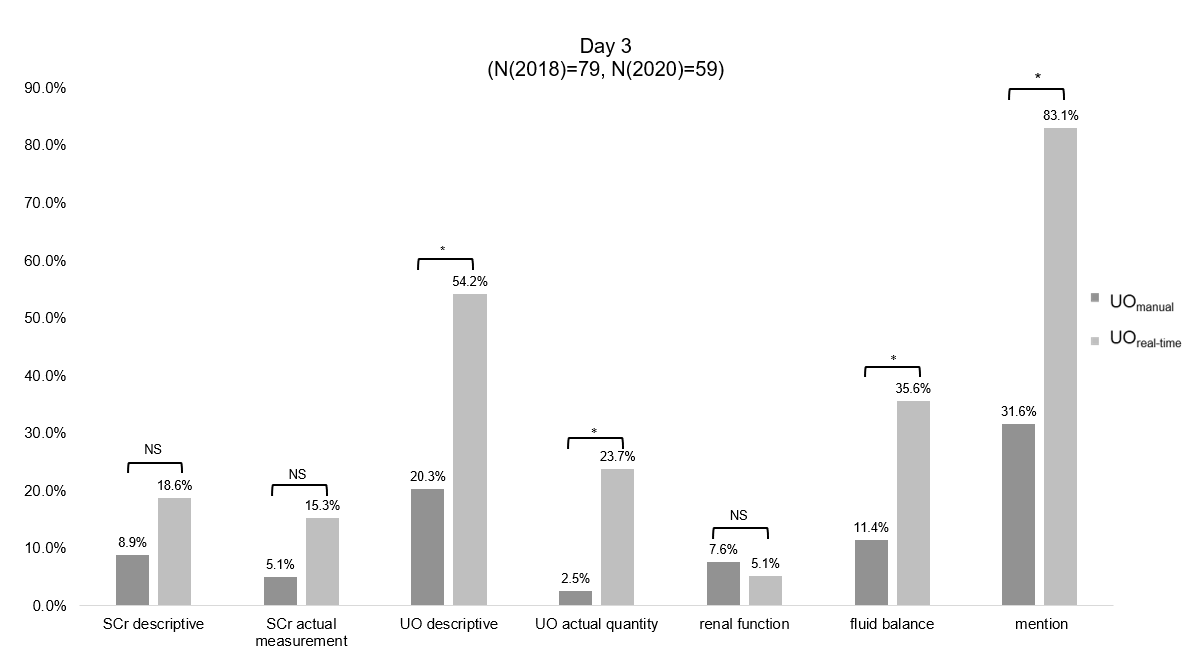


Supplementary Fig 1b.


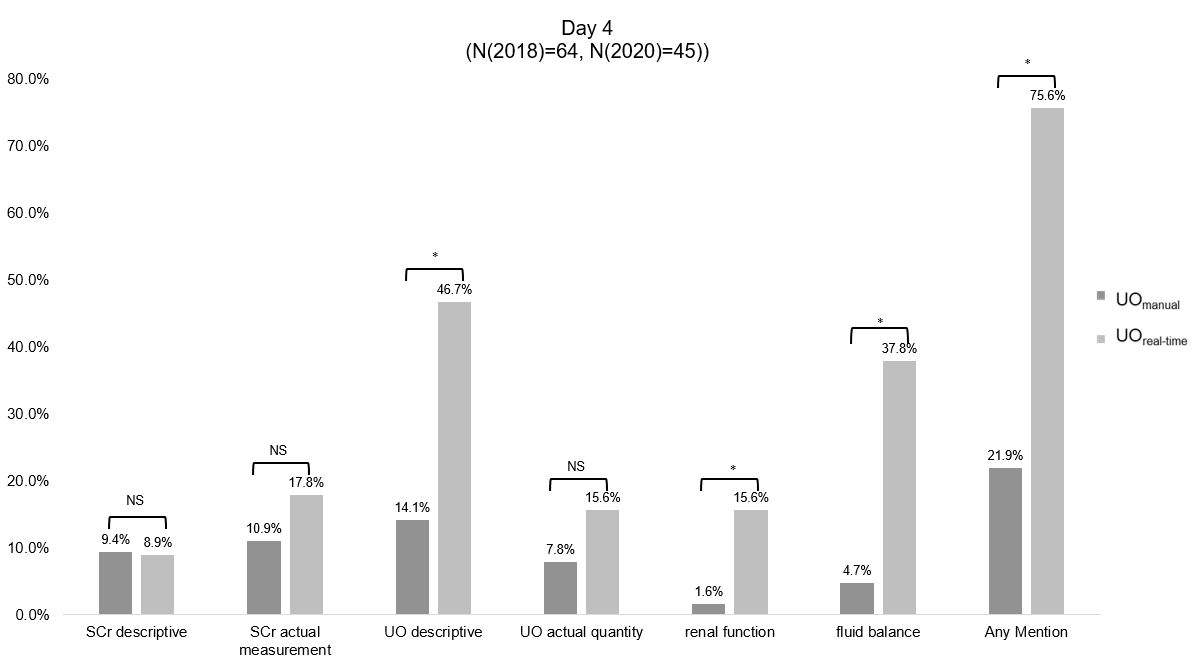


Supplementary Fig 1c.


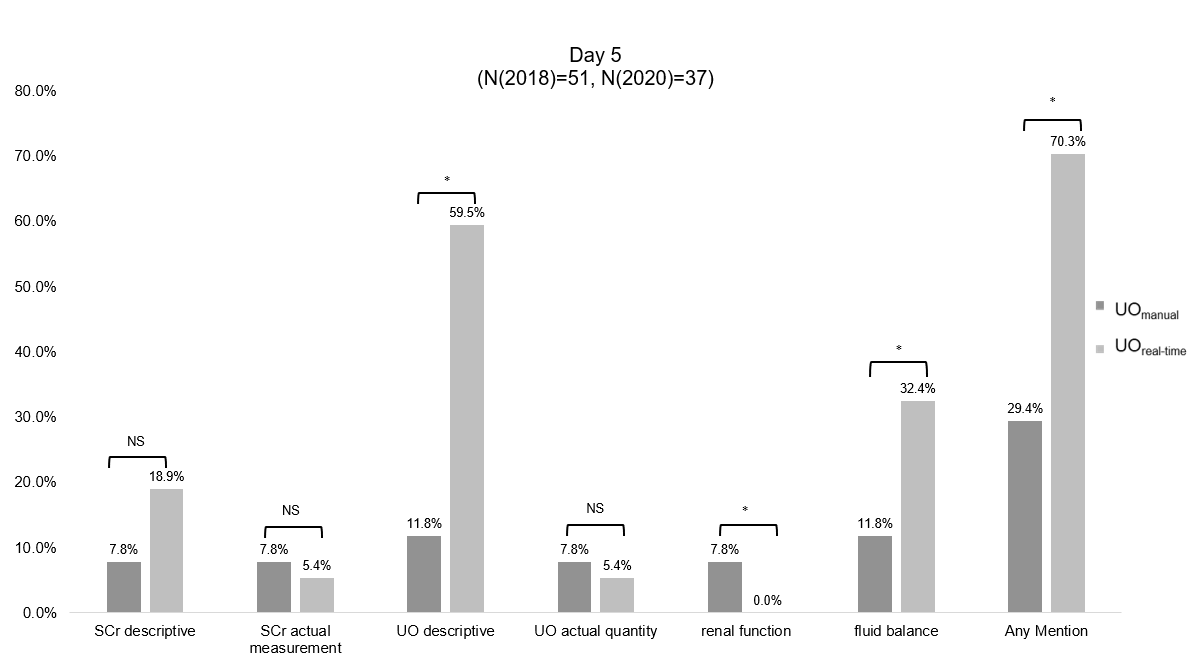


Supplementary Fig 1d.


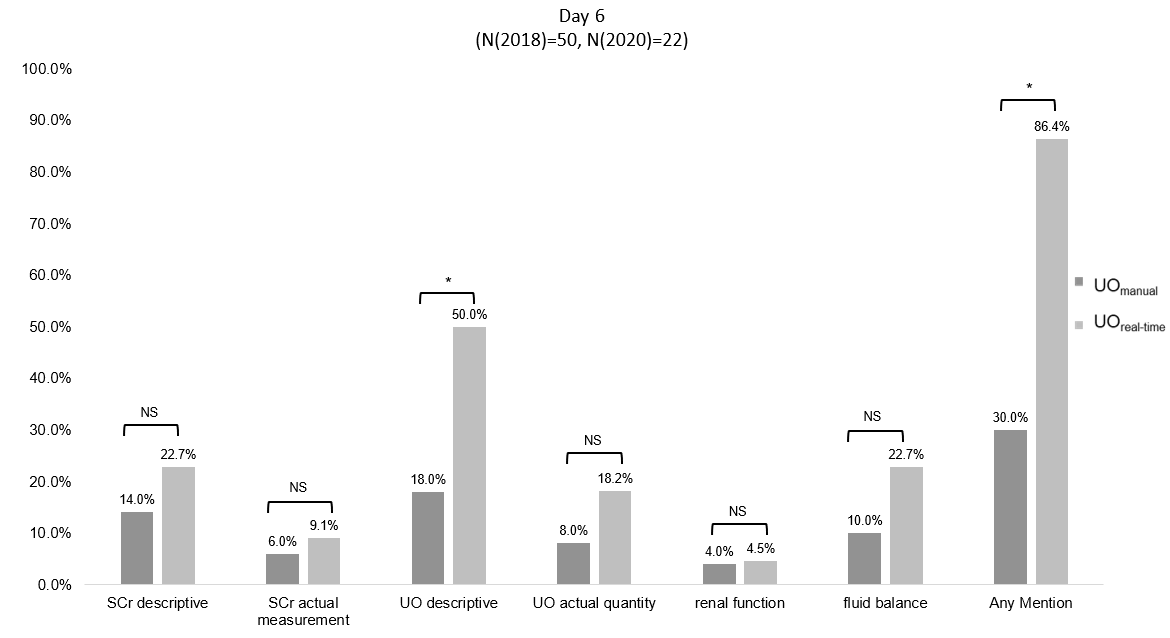


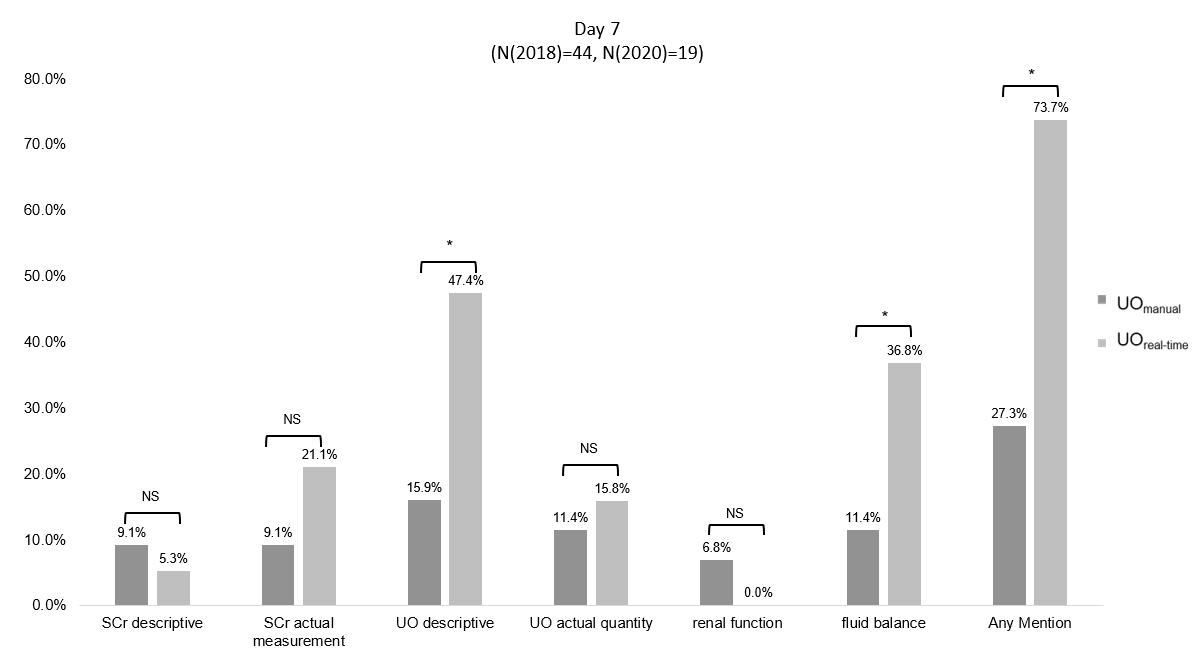
Supplementary Fig 1e.
